# Secrets of the *MIR172* family in plant development and flowering unveiled

**DOI:** 10.1371/journal.pbio.3001099

**Published:** 2021-02-08

**Authors:** Bailong Zhang, Xuemei Chen

**Affiliations:** Department of Botany and Plant Sciences, Institute of Integrative Genome Biology, University of California, Riverside, California, United States of America

## Abstract

In plants, conserved microRNAs tend to be encoded by gene families with multiple members. This Primer explores the implications of two recent studies that interrogated the functions of the five-member MIR172 family in Arabidopsis, revealing complexities and intricacies of the gene regulatory networks underlying floral transition.

## Teaser

In plants, conserved microRNAs (miRNAs) tend to be encoded by gene families with multiple members. Two recent studies interrogated the functions of the 5-member *MIR172* family in *Arabidopsis* and revealed complexities and intricacies of gene regulatory networks underlying floral transition.

MicroRNAs (miRNAs) are 20- to 24-nucleotide long, noncoding RNAs that regulate target genes at posttranscriptional levels [1]. Plants maximize their fitness by employing miRNAs to regulate various developmental processes, particularly in coordination with environmental cues [1]. Plant miRNAs are especially suited as developmental regulators as they, through a high degree of sequence complementarity, target a small number of genes (usually belonging to a gene family) at the nodes of gene regulatory networks. In addition, their own accumulation is subjected to spatial and temporal regulation through *MIR* gene promoters. *MIR* genes undergo dynamic birth and loss during evolution, such that only tens of miRNAs are conserved in seed plants [2,3]. Owing to whole genome, segmental, and gene duplication events in land plant evolution, conserved miRNAs tend to belong to gene families, in which identical or nearly identical mature miRNA sequences are encoded by different *MIR* genes [4]. Partial redundancy together with subfunctionalization and/or neofunctionalization among *MIR* family members potentially enhances regulatory dimensions and robustness. Do *MIR* gene family members exhibit different spatiotemporal patterns in expression? Do they integrate different environmental signals to impact developmental processes? Do they act on the same or different sets of targets? These questions are still largely unknown for most *MIR* gene families in plants. Two recent studies [5,6] provided insights into some of these questions by interrogating the *Arabidopsis* 5-member *MIR172* family, a miRNA family conserved in spermatophytes [3].

Several approaches have been routinely used to determine the biological functions of miRNAs, but none can readily reveal the functions of individual *MIR* gene family members. First, individual *MIR* genes can be over/ectopically expressed, and the phenotypic consequences are used to infer the biological functions of the miRNA. However, the approach itself alters the natural patterns of *MIR* gene expression and thus cannot confidently capture the endogenous functions of *MIR* genes. Second, expression of a miRNA-resistant target using its own promoter is a powerful approach to uncover the consequences when a particular target gene is released from miRNA regulation. However, this only reflects partial functions of a miRNA, as other targets are still being repressed by the miRNA. A third useful approach is target mimicry—the expression of an RNA with a non-cleavable miRNA target site [7]. The target mimic RNA leads to the degradation of the cognate miRNA or serves as a sponge to prevent the miRNA from accessing its natural targets. However, none of the approaches above can separate the regulatory contributions of individual *MIR* gene members. Finally, loss-of-function approaches can be used to study *MIR* genes as for protein-coding genes by taking advantage of the existing transfer DNA (T-DNA) mutants. Given the small size of precursor miRNAs (approximately 200 nucleotides), T-DNA insertion mutants in all *MIR* gene family members may not be readily available. Recently, gene editing technology based on clustered regularly interspaced short palindromic repeats (CRISPR)-Cas9 enables the construction of loss-of-function mutants for *MIR* genes and provides an opportunity to systematically interrogate the functions of individual members of *MIR* gene families.

Flowering at an appropriate time is critical for plants’ reproductive success and is thus regulated by sophisticated gene networks that monitor and respond to environmental changes and endogenous cues. In *Arabidopsis*, miR172 targets 6 members of the *APETALA2* (*AP2*)-like family of transcription factor genes and plays critical roles in flowering time control [8,9]. However, little was known about how the presence of 5 *MIR172* genes influences the gene regulatory networks underlying flowering. In the 2 studies published recently, the authors generated loss-of-function mutants in each of the 5 *MIR172* genes and analyzed their developmental and particularly flowering time phenotypes. Moreover, they generated different combinations of higher-order *mir172* mutants and further explored functional redundancy and/or specificity among the 5 genes. These studies, together with the use of reporter genes to monitor the expression patterns of each gene, demonstrate that the *Arabidopsis MIR172* gene family members have divergent and common functions in integrating various endogenous and exogenous cues to execute flowering [5,6].

Upon floral induction, the shoot apical meristem (SAM) mainly produces flowers instead of leaves. Plants regulate flowering time via integration of environmental signals with endogenous cues. Genetic pathways that regulate flowering by responding to both environmental changes, such as seasonal changes in day length (photoperiod), and endogenous developmental information, such as plant age, have been elucidated [10]. These pathways converge on the floral integrators *FLOWERING LOCUS T* (*FT*) and *SUPPRESSOR OF OVEREXPRESSION OF CONSTANS 1* (*SOC1*), whose induction leads to flowering [10].

**Fig 1:**
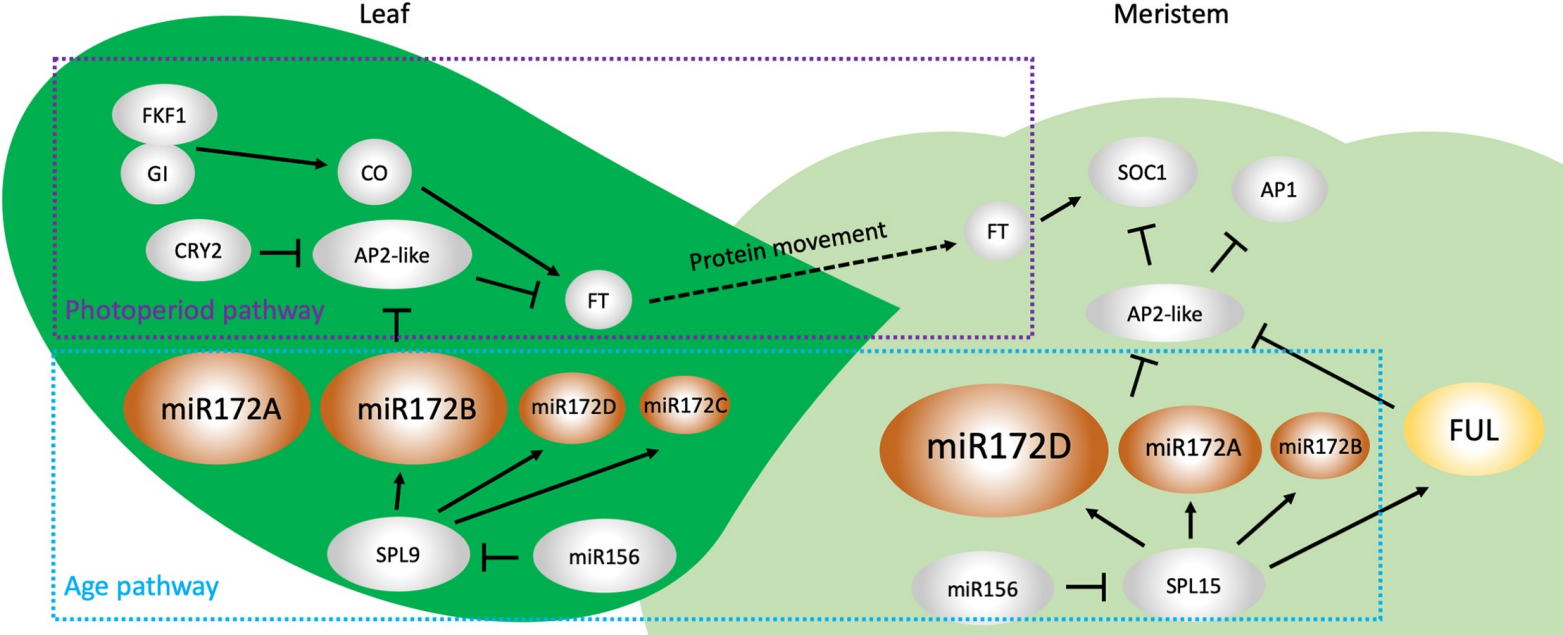
Model of *MIR172* genes in regulating flowering time in leaves and the SAM. In leaves, *MIR172A* and *MIR172B* are the major *MIR172* genes that promote flowering under LD. In the SAM, *MIR172D* is the major *MIR172* gene that promotes flowering under SD. Sizes of the ovals representing *MIR172* members approximate their activity levels. AP1, APETALA1; AP2, APETALA2; AP2-like, AP2-like proteins; CO, CONSTANS; CRY2, CRYPTOCHROME2; FKF1, FLAVIN-BINDING KELCH REPEAT F-BOX1; FT, FLOWERING LOCUS T; FUL, FRUITFUL; GI, GIGANTEA; LD, long days; SAM, shoot apical meristem; SD, short days; SMZ, SCHLAFMUTZE; SNZ, SCHNARCHZAPFEN; SOC1, SUPPRESSOR OF OVEREXPRESSION OF CONSTANS1; SPL, SQUAMOSA PROMOTER BINDING LIKE; TOE1, TARGET OF EAT1; TOE2, TARGET OF EAT2; TOE3, TARGET OF EAT3.

The photoperiod pathway (Fig 1) senses day length in leaves through a signaling cascade involving GIGANTEA (GI), FLAVIN-BINDING KELCH REPEAT F-BOX 1 (FKF1) and the transcription factor CONSTANS (CO) [10]. CO promotes flowering by activating the transcription of *FT* in the leaf vasculature [10]. AP2-like proteins harboring a transcriptional repression Ethylene-responsive element binding-factor-associated Amphiphilic Repression (EAR)-like motif interact with CO and repress *FT* expression [11], and this interaction was recently shown to be modulated by the blue light receptor CRYPTOCHROME2 (CRY2) [12]. The FT protein moves through the phloem to the SAM where it activates the floral developmental program. The 2 recent studies on the *MIR172* family [5,6] demonstrated that *MIR172A* and *MIR172B* play major roles in promoting flowering under long days (LD) (Fig 1). Consistently, both genes are expressed in the leaf vasculature under LD.

Under short days (SD), the age pathway (Fig 1) plays a major role in flowering. Expression of the *SQUAMOSA PROMOTER BINDING LIKE* (*SPL*) family genes, which promote flowering, increases as plants age [10,13]. *SPL* genes are the targets of miR156, whose levels are high in young plants and progressively decrease as plants develop [10,13]. The levels of miR172 mirror those of miR156 as *MIR172* genes are activated by *SPL* genes [13]. Under SD, the repression of *AP2*-like genes by miR172 induces the expression of *SOC1*, as well as *FRUITFUL* (*FUL*) and *APETALA1*, in the SAM to promote flowering. *MIR172D* functions as the major *MIR172* family member in the SAM to promote flowering under SD, with *MIR172A* and *MIR172B* playing a minor role (Fig 1). Consistently, *MIR172D* is highly expressed in the SAM.

The studies also provided insights into the intricacies of the gene regulatory networks underlying flowering (Fig 1). In leaf vasculature, the promoter activities of *MIR172B* and *C*, but not those of *MIR172A*, were reduced in an *spl9* mutant. Thus, *MIR172* members respond differently to this upstream activator. In the SAM, it was shown previously that miR172 levels are greatly reduced in an *spl15* mutant [14]. Consistently, Ó’Maoiléidigh and colleagues showed that the promoter activities of *MIR172A-D* were all increased in plants expressing miR156-resistant *SPL15*, suggesting that *SPL15* activates these 4 genes [5]. Intriguingly, the late flowering phenotype of an *spl15* mutant is strongly enhanced by mutations in either *MIR172A* or *MIR172B*, suggesting that another gene(s) activates the expression of these *MIR172* genes in the absence of *SPL15*. In addition, the relationship between *MIR172* and *FUL*, which repress the same *AP2*-like genes at the posttranscriptional and transcriptional levels, respectively, was also studied. Under both LD and SD conditions, *MIR172* genes and *FUL* appear to act in parallel to promote the expression of the floral integrators. The work also begins the analysis of the effects of *MIR172* genes on *AP2* expression [5], but the regulatory relationships among the 5 *MIR172* genes and the 6 *AP2*-like target genes remain largely unexplored. With mutants in each *MIR172* family member, it is now possible to discern how *MIR172* genes contribute individually or collectively to target gene repression. In summary, the 2 studies demonstrate that the redundancy and specificity of *MIR172* family members increase the robustness and dimensions of the gene regulatory networks underlying flowering.

## Abbreviations

AP2: APETALA2
CO: CONSTANS
CRISPR: clustered regularly interspaced short palindromic repeats
CRY2: CRYPTOCHROME2
EAR: Ethylene-responsive element binding-factor-associated Amphiphilic Repression
FKF1: FLAVIN-BINDING KELCH REPEAT F-BOX 1
FUL: FRUITFUL
FT: FLOWERING LOCUS T
GI: GIGANTEA
LD: long days
miRNA: microRNA
SAM: shoot apical meristem
SD: short days
SOC1: SUPPRESSOR OF OVEREXPRESSION OF CONSTANS 1
SPL: SQUAMOSA PROMOTER BINDING LIKE
T-DNA: transfer DNA

## References

[1] Chen X. Small RNAs and their roles in plant development. Annu Rev Cell Dev Biol. 2009;25:21–44. 10.1146/annurev.cellbio.042308.113417 19575669 PMC5135726

[2] Fahlgren N, Howell MD, Kasschau KD, Chapman EJ, Sullivan CM, Cumbie JS, et al High-throughput sequencing of *Arabidopsis* microRNAs: evidence for frequent birth and death of MIRNA genes. PLoS ONE. 2007;2(2):e219 10.1371/journal.pone.0000219 17299599 PMC1790633

[3] Luo Y, Guo Z, Li L. Evolutionary conservation of microRNA regulatory programs in plant flower development. Dev Biol. 2013;380(2):133–44. 10.1016/j.ydbio.2013.05.009 23707900

[4] Li A, Mao L. Evolution of plant microRNA gene families. Cell Res. 2007;17(3):212–8. 10.1038/sj.cr.7310113 17130846

[5] Ó’Maoiléidigh D, van Driel A, Singh A, Sang Q, Bec N, Vincent C, et al Systematic analyses of the *MIR172* family members of Arabidopsis define their distinct roles in regulation of *APETALA2* during floral transition. PLoS Biol. 2021. 10.1371/journal.pbio.3001043 PMC7853530 33529186

[6] Lian H, Wang L, Ma N, Zhou C, Han L, Zhang T, et al Redundant and specific roles of individual *MIR172* genes in plant development. PLoS Biol. 2021. 10.1371/journal.pbio.3001044 PMC7853526 33529193

[7] Franco-Zorrilla JM, Valli A, Todesco M, Mateos I, Puga MI, Rubio-Somoza I, et al Target mimicry provides a new mechanism for regulation of microRNA activity. Nat Genet. 2007;39(8):1033–7. 10.1038/ng2079 17643101

[8] Aukerman MJ, Sakai H. Regulation of flowering time and floral organ identity by a MicroRNA and its *APETALA2*-like target genes. Plant Cell. 2003;15(11):2730–41. 10.1105/tpc.016238 14555699 PMC280575

[9] Chen X. A microRNA as a translational repressor of *APETALA2* in *Arabidopsis* flower development. Science. 2004;303(5666):2022–5. 10.1126/science.1088060 12893888 PMC5127708

[10] Fornara F, de Montaigu A, Coupland G. SnapShot: Control of flowering in *Arabidopsis*. Cell. 2010;141(3):550, e1–2. 10.1016/j.cell.2010.04.024 20434991

[11] Zhang B, Wang L, Zeng L, Zhang C, Ma H. *Arabidopsis* TOE proteins convey a photoperiodic signal to antagonize CONSTANS and regulate flowering time. Genes Dev. 2015;29(9):975–87. 10.1101/gad.251520.114 25934507 PMC4421985

[12] Du SS, Li L, Li L, Wei X, Xu F, Xu P, et al Photoexcited cryptochrome2 interacts directly with TOE1 and TOE2 in flowering regulation. Plant Physiol. 2020;184(1):487–505. 10.1104/pp.20.00486 32661061 PMC7479908

[13] Poethig RS. The past, present, and future of vegetative phase change. Plant Physiol. 2010;154(2):541–4. 10.1104/pp.110.161620 20921181 PMC2949024

[14] Hyun Y, Richter R, Vincent C, Martinez-Gallegos R, Porri A, Coupland G. Multi-layered regulation of SPL15 and cooperation with SOC1 integrate endogenous flowering pathways at the *Arabidopsis* shoot meristem. Dev Cell. 2016;37(3):254–66. 10.1016/j.devcel.2016.04.001 27134142

